# Esterified Hyaluronic Acid Matrix in Lower Extremity Reconstruction With Exposed Tendon and Bone: A Retrospective Review

**DOI:** 10.1093/jbcr/iraa044

**Published:** 2020-04-18

**Authors:** Steven D Kozusko, Mahmoud Hassouba, David M Hill, Xiangxia Liu, Kalyan Dadireddy, Sai R Velamuri

**Affiliations:** 1 Division of Plastic Surgery, Cooper University Hospital, Camden, New Jersey; 2 Department of Plastic Surgery, University of Tennessee Health Science Center, Memphis; 3 University of Tennessee Health Science Center, Memphis; Firefighters’ Regional Burn Center, Memphis, Tennessee

## Abstract

Lower extremity wounds with exposed bone and tendon often need coverage to allow the underlying tissue to regenerate prior to skin graft. The surgeon is limited in his or her choices to augment tissue regeneration in these types of complicated cases; for instance, autologous skin should not be placed on exposed bone or tendon and is at risk for contracture when placed over the joints. Therefore, novel technologies are necessary to provide a scaffolding for tissue to regenerate and allow for a successful graft. One such technology is an esterified hyaluronic acid matrix (eHAM), which can provide a proper scaffold for endothelial cell migration and aid in angiogenesis. The eHAM is made of two layers: a layer of hyaluronic acid covered with a silicone layer. In this retrospective chart review, we describe our usage of eHAM to provide scaffolding for tissue regeneration prior to grafting in 15 cases of complicated lower extremity wounds with exposed bone and tendon. The average patient age was 45.8 years, and all patients had multiple medical comorbidities, such as poorly controlled diabetes mellitus, hypertension, and nicotine addiction. Patient wound types were diverse, including traumatic wounds, chronic diabetic foot ulcers, and thermal or electric burns. Thirteen of the 15 cases were treated successfully with eHAM. In these cases, definitive coverage with split-thickness skin grafting was effective and limb salvage was successful. In the 13 successful cases, the mean time to split-thickness skin graft was 22.9 ± 7.0 days. All patients continue to do well at follow-up (ranging from 6 to 48 weeks), with minimal complications reported. Given the success rate with eHAM in this challenging population, we conclude that eHAM can be a treatment option for similar cases.

Complex wounds require coverage to prevent infection, subsequent sepsis, and mortality. Damage to the skin barrier can result in fluid loss and shock secondary to volume loss. Tissue desiccation can increase the zone of injury. Dermal coverage is required to reduce infection in both burn and chronic wounds.^1^

Coverage of complex wounds includes many options. Autologous skin is one of the gold standards for coverage of wounds, as it can be widely meshed and will not undergo rejection from the host. However, it should not be placed on exposed bone or tendon and is at risk for contracture when placed over joints.^2^ Human allograft, in a cryopreserved form, is another option for wound coverage. It can be stored for long durations, conforms to wounds, prevents water loss, and functions as a barrier to organisms.^3^ However, it usually undergoes rejection over the course of a few weeks and undergoes necrosis. One major breakthrough in coverage of complex wounds came with the development of regenerative dermal matrix products.^4^ One commonly used option consists of a bilaminate template with an outer protective layer, made from silicone that sits atop a dermal matrix.^5^ The latter is composed of bovine collagen and chondroitin-6-sulfate. The silicone layer is removed in 3 to 4 weeks when thin split-thickness skin grafting (STSG) is completed. This skin substitute interacts with the wound bed without causing inflammation or rejection. Host fibroblasts scaffold with the substitute and angiogenesis occurs.^6^ The end result is a viable neodermis and definitive coverage with an STSG. Use of this technology is limited by cost and infection risk.

An esterified hyaluronic acid matrix (eHAM) can provide a suitable scaffold for endothelial cell migration and generation of engineered vascular grafts.^7^ This product has been used in the treatment of complex wounds.^8^ The eHAM consists of two layers: one layer consists of a controlled release form of hyaluronic acid (HA) in a matrix that is covered with a silicone layer. The dermal-like matrix maintains moisture in the wound bed, allowing for capillary ingrowth and cellular invasion. Angiogenesis and dermal regeneration with endothelial cells and fibroblasts promote tissue regeneration.^9^ The silicone layer acts as a barrier to organisms and subsequent infection. The benefits of eHAM include a moist healing environment, ease of application and removal, and prevention of infection.

As the reconstructive surgeon proceeds up the ladder, local, regional, and free flaps become treatment options. However, the lower extremity is a challenging area to reconstruct with only local and regional flaps. Often, free flaps are implemented in the lower extremity.^10^ When multiple medical comorbidities exist, the patient may be a poor candidate for microsurgical reconstruction, and alternative treatment modalities must be considered. The purpose of this study is to present the efficacy of an eHAM in lower extremity wounds with critically exposed tendon and/or bone. The aim is to add one technique to the armamentarium of reconstructive surgeons dealing with complex wounds.

## MATERIALS AND METHODS

Expedited review for the case study was granted by the institutional review board (IRB). After obtaining IRB approval, we performed a retrospective review of patients treated with an eHAM (Hyalomatrix^®^, Medline Industries, Inc., Northfield, IL) at a single regional burn center in the past year. Inclusion criteria included adults aged 18 or older with a lower extremity wound(s), treated with eHAM, and who were not candidates for free flap reconstruction. Exclusion criteria were wounds in areas other than the lower extremity or treatment with other dermal substitutes. The patients’ charts were reviewed to assess demographics, time to graft, follow-up duration, mechanism, comorbidities, infections, outcomes, and complications. Additionally, photographic images were obtained from the burn center camera for each patient to assess the clinical progression of healing. Results were analyzed using Sigma Plot 11.2 and reported as descriptive statistics. Shapiro–Wilk test was used to test for normality.

## RESULTS

This series consisted of 15 patients with multiple medical comorbidities and exposed critical structures: 13 were successfully treated with eHAM, and in two patients the eHAM did not adhere to the wound bed. Both failures occurred in patients with exposed bone and multiple comorbidities including diabetes mellitus and hypertension.

The following data are based on all 15 patients. The mean patient age was 45.8 ± 18.4 years. The mean time to split-thickness skin graft was 22.9 ± 7.0 days in the 13 successful cases. The median follow-up was 12 weeks, with a range of 6 to 48 weeks. Six patients sustained an injury due to either a thermal or an electrical burn, and three patients had wounds due to diabetic complications. Eleven patients were active smokers. Nine patients had hypertension and five patients had either end-stage renal disease or chronic kidney disease (see Table 1 for more information on patients). Coverage included exposed bone (ie, calcaneus or tibia), Achilles tendons, and dorsal foot extensor tendons. One patient received bedside application of eHAM. This patient was not medically stable for the operating room and general anesthesia. One patient had the eHAM applied at the bedside, instead of the operating room. This patient had granulated a majority of the wound but had a small 1 cm by 1 cm area needing a second application. Below we present three representative cases in detail.

**Table 1.** Demographics and etiologies for case study patients

Patient	Outcome	Structure	Age	Sex	Days to Graft	Weeks to Follow-up	Pathology	Comorbidities
1	Success	Tendon	49	F	25	8	Burn	CHF, ESRD, DM, HTN, Smoker
2	Success	Bone	44	M	28	12	Diabetic ulcer	ESRD, DM, HTN, Smoker
3	Success	Bone	50	F	26	48	Diabetic ulcer	DM
4	Success	Tendon	31	M	14	6	Burn with exposed Achilles	Smoker
5	Success	Bone	35	M	36	36	Burn with exposed bone	HTN, AKI on CRRT, PE, DVT, PVD, 60% TBSA, Smoker
6	Success	Tendon	53	M	14	12	Burn with exposed Achilles	HTN, DM, Schizophrenia, Depression, Smoker
7	Success	Bone/Joint	29	M	34	28	Electrical burn with osteomyelitis	Smoker
8	Success	Tendon	18	M	18	24	Crush injury	None
9	Success	Tendon	72	M	22	12	Scald burn	DM, HTN, CKD
10	Success	Tendon	83	F	22	8	Chronic wound	AF, COPD, PVD
11	Failure	Bone	48	F	N/A	N/A	Wound with exposed bone	DM, HTN
12	Success	Bone/Joint	46	M	19	12	Crush injury with exposed joint	Smoker
13	Success	Bone	35	M	16	16	Exposed bone	HTN, CKD, PE, DVT, PVD, Smoker
14	Failure	Bone	70	F	N/A	N/A	Diabetic ulcer with osteomyelitis	DM, HTN
15	Success	Bone/Joint	24	M	24	12	Motor vehicle collision and abrasions	Smoker

*CHF*, congestive heart failure; *ESRD*, end-stage renal disease; *DM*, diabetes mellitus; *HTN*, hypertension; *AKI*, acute kidney injury; *CRRT*, continuous renal replacement therapy; *PE*, pulmonary embolism; *PVD*, peripheral vascular disease; *TBSA*, total body surface area; *CKD*, chronic kidney disease; *AF*, atrial fibrillation; *COPD*, chronic obstructive pulmonary disease.

### Case Examples

#### Patient 5

**Figure 1.**
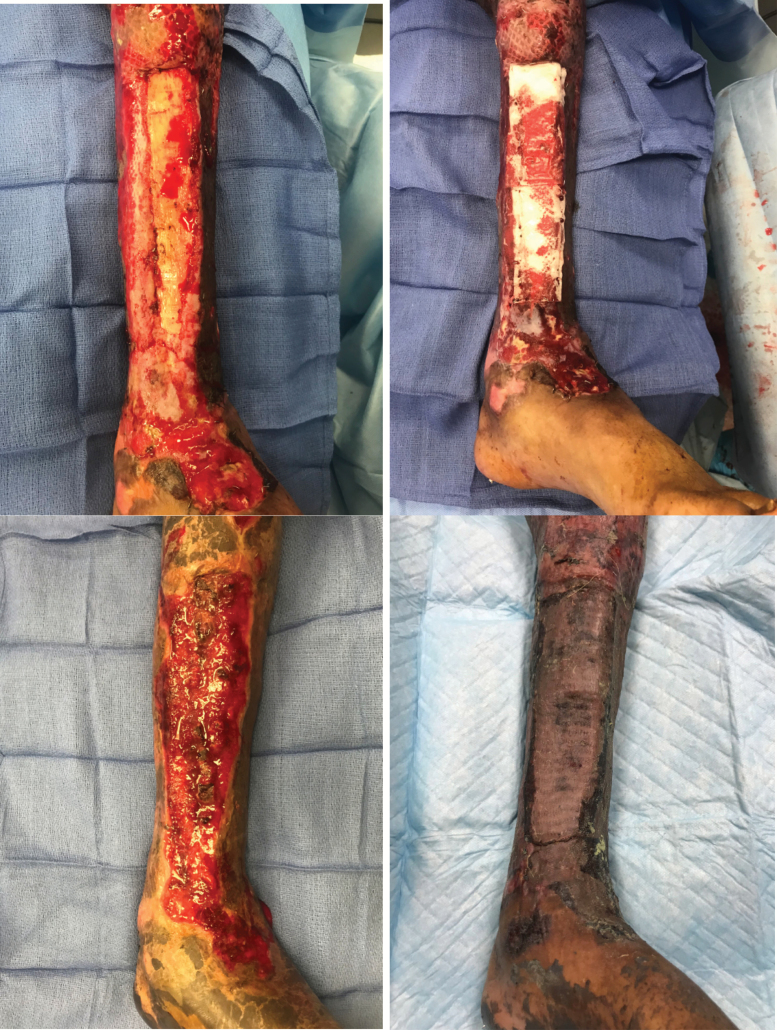
Burn with more than 10 cm of the exposed tibia. Exposed tibia after failed Integra (top left); application of eHAM (top right); granulation tissue at day 36 (bottom left); and healed STSG at 36 weeks follow-up (bottom right). *eHAM*, esterified hyaluronic acid matrix; *STSG*, split-thickness skin grafting.

The patient is a 35-year-old man with 60% total body surface area (TBSA) third-degree burns involving upper and lower extremities (Figure 1). His lower extremity burn wounds extended to the bone. Multiple medical comorbidities were present, including hypertension, acute kidney injury requiring dialysis, pulmonary embolism, and active deep vein thrombosis to left lower extremity, peripheral vascular disease, and hypertension. He underwent multiple reconstructive procedures including gastrocnemius/tibialis anterior muscle flap for upper 1/3 exposure, but failed PriMatrix application to the lower two third of the wound. The patient successfully underwent the application of eHAM with two different applications. The first application resulted in granulation tissue over a majority of the exposed bone. With a small area of exposed bone remaining, the decision was made to apply a second small piece of the matrix. Healthy exuberant granulation tissue was present over the bone within 2 weeks of application. The entire bony defect was covered by granulation tissue within 5 weeks. Skin grafts were stable and patient ambulatory with a custom fit orthotic shoe at 6 months follow-up.

#### Patient 7

**Figure 2.**
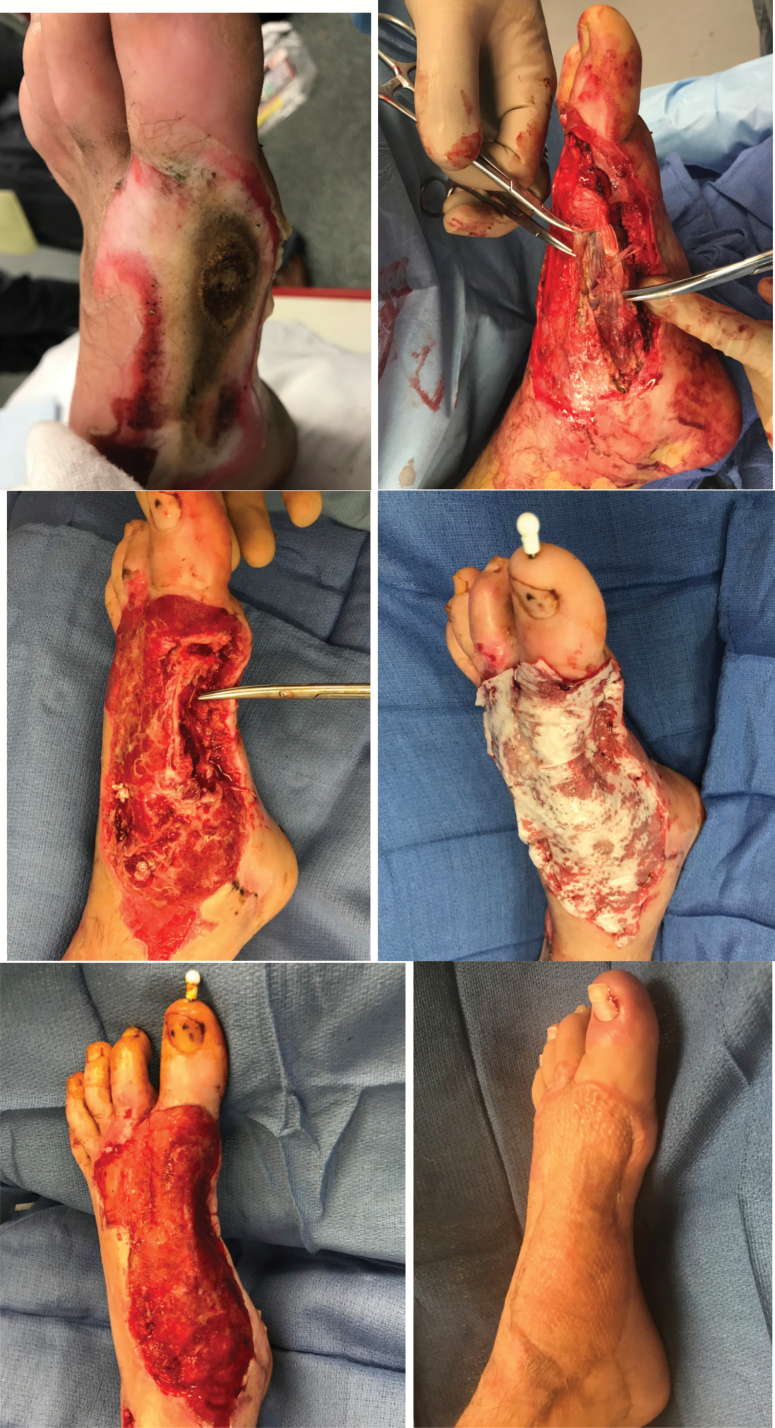
Electrical burn with metatarsal osteomyelitis. Initial electrical burn presentation (top left); necrotic first metatarsal (top right); debridement of metatarsal (middle left); application of eHAM (middle right); granulation tissue at day 34 (bottom left); and healed STSG at 28 weeks follow-up (bottom right). *eHAM*, esterified hyaluronic acid matrix; *STSG*, split-thickness skin grafting.

The patient is a 29-year-old man with an electrical injury to the right foot. Initial debridements revealed a necrotic first metatarsal (Figure 2). The patient developed acute osteomyelitis to the foot. Limb salvage was successful using skin substitute eHAM with two different applications. Time to grafting was 5 weeks after two applications. A second application involved coverage of a limited area to the first metatarsal as most of the wound had granulated. Skeletal stabilization was also completed to immobilize the right first metatarsophalangeal joint using K wires. No open wounds were present on follow-up at 6 months. The patient is ambulating with a custom fit orthotic shoe.

#### Patient 4

**Figure 3.**
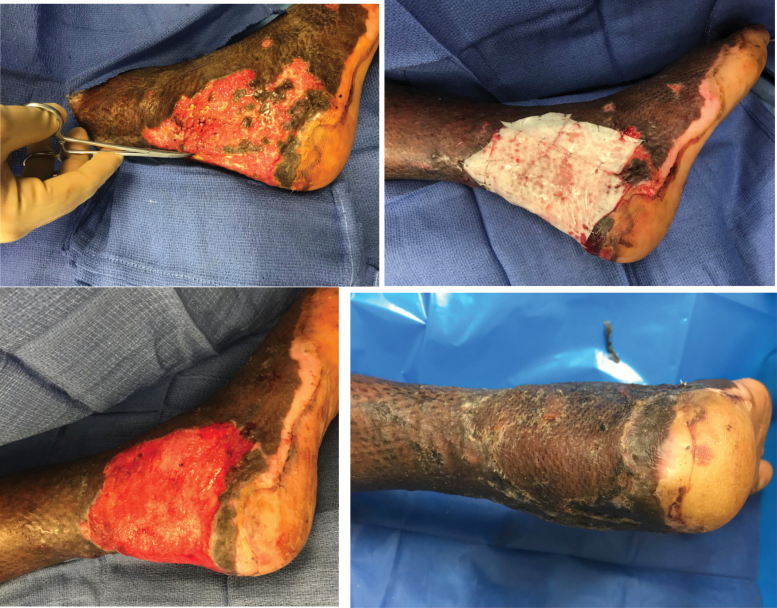
Burn with exposed Achilles tendon. Achilles tendon exposed (top left); application of eHAM (top right); granulation tissue at day 14 (bottom left); and healed STSG at 6 weeks follow-up (bottom right). *eHAM*, esterified hyaluronic acid matrix; *STSG*, split-thickness skin grafting.

The patient is a 31-year-old man with 30% TBSA third-degree burns to his body (Figure 3). After undergoing skin grafting, he developed exposure to his right foot Achilles tendon. eHAM was used and secured with Acticoat silver dressing. Healthy granulation tissue was present over tendon within 2 weeks. The wound bed from eHAM was superficially excised and grafted. The patient had a 100% skin graft take with no breakdown at 6 weeks of follow-up.

## DISCUSSION

Complex cases of wound reconstruction include a variety of factors that prevent immediate definitive coverage using autologous tissue. Major burns limit the availability of skin for grafting.^1^ Patient comorbidity factors including diabetes mellitus, peripheral neuropathy, chronic renal disease, and impaired mobility secondary to disease complications may preclude free tissue transfer.^11^

Cost is a concern that has driven surgeons to develop and use less expensive tissue substitutes.^12^ Medicare lists Integra as a high-cost skin substitute on its outpatient prospective payment system cost category assignment, while listing eHAM agents as low cost. This is the most accurate comparison of cost, as openly transparent cost information is difficult to obtain. At this institution, a 2 in. by 2 in. piece of Integra dermal regeneration template (25 sq cm) will cost more than $1000. The cost of eHAM for a 5 cm by 5 cm piece of matrix (25 sq cm) costs hundreds of dollars. These numbers are relative, as each institution’s cost for a skin substitute will vary based on the size of purchase and consignment.

When critical structures, such as bone and tendons are exposed, dermal coverage is often needed prior to skin grafting.^13^ Dermal coverage is necessary to prevent infection, desiccation, and osteomyelitis. eHAM is a modality that creates a granulation bed that covers critical structures, allowing for subsequent skin grafting in a properly selected and challenging population. This is the first series on eHAM that focuses on the lower extremity and coverage of exposed tendon and bone.

HA is a component of the human extracellular matrix that is highly concentrated in the skin. It is a glycosaminoglycan that functions to allow hydration and modulation of microenvironments within cells.^14^ The eHAM has a matrix layer in contact with the wound and a silicone layer as an outside barrier. The inner layer is a biodegradable matrix composed of HA. The matrix and HA maintain moisture, provide a scaffold for fibroblasts and endothelial cells, conform to the complex wound, and promote angiogenesis.^7,9^ These factors are important in the healing process, until a regenerated dermis is present. The eHAM can prolong the half-life of HA and provide a controlled rate of biodegradation of the matrix that remains at the wound site in order to promote healing.^15^

The eHAM has been used successfully in a series of 300 pediatric burn patients.^16^ They were treated with eHAM application and subsequent skin grafting. All of the patients sustained burns, but no patients were treated for exposed tendon or bone. Pediatric patients are relatively healthy in comparison to the cohort presented in our series. Another series shows that eHAM provided a moist environment, prevented eschar formation, and promoted painless granulation tissue formation.^14^ This study evaluated only partial-thickness burns of pediatric patients.

A retrospective review on the use of eHAM was performed in 11 burn centers throughout Italy.^1^ In the 2-year study period, the eHAM was used in 57 patients with burns involving less than 50% TBSA. This study demonstrated eHAM utility in both partial-thickness and full-thickness burns. Wound closure was achieved after a median of 29 days in partial-thickness and 39 days in full-thickness burns. The results of this study show that eHAM can help bridge both partial- and full-thickness wounds to definitive grafting. Exposed tendons and bone were not addressed in this study.

One study demonstrated eHAM’s efficacy in treating 10 patients with hypertrophic scars and keloids.^17^ The protocol involved excision of the pathology with subsequent application of eHAM. On the 28th postoperative day the patients underwent STSG. All patients healed without recurrence of the respective pathology. Though a small series, this demonstrates the range of efficacy of eHAM. Another study on six patients with hypertrophic scars demonstrated similar efficacy.^18^ All patients underwent surgical excision of the scar with the placement of an eHAM. STSG was performed at a mean time of 19 days after application. They reported two patients with loss of STSG, a potential complication of eHAM reconstruction.

Congenital syndactyly is often treated with full-thickness skin grafts after the release of the pathology involving the digits. A study evaluated the treatment of 23 children with syndactyly using eHAM.^19^ Unique to this study, none of the patients required subsequent skin grafting. The open areas after syndactyly release were treated with an eHAM for 3 weeks followed by dressing care. Favorable web creep results were observed in all patients. This study demonstrates a situation where eHAM can be used without the need for a subsequent STSG.

An exposed tendon is one clinical situation that usually requires complex reconstructive considerations including local, regional, or free flaps. The literature has a paucity of information regarding the coverage of tendon or bone with eHAM. One review of eHAM in burn patients reported a study that used eHAM with platelet-rich plasma (PRP) for treating tendon exposure.^20^ The authors of this study treated surgically debrided, burn wounds with PRP followed by eHAM. They noted the severity of open wounds being reduced with the combination therapy. This study did not evaluate eHAM alone or in the setting of complex wounds related to other pathology.

The eHAM can be used as a dermal regenerative matrix in complex wound cases as a bridge to definitive coverage with autologous skin grafting. This study has demonstrated successful coverage of tendons, bones, and chronic wounds in mean time of 3 to 4 weeks in a challenging population. There are a few details of this study not otherwise reported well in the literature. First, there was a high success rate in patients not expected to undergo successful limb salvage. Second, the patients in this study have multiple medical comorbidities including nicotine use, poorly controlled diabetes mellitus, hypertension, congestive heart failure, and older age. This precluded this cohort from microsurgical coverage of exposed tendon and bone. Thus, the main benefit of eHAM is that it can be used in a patient who is not a reconstructive candidate and has no local reconstructive options. Third, this series focuses on wounds of the lower extremity from multiple etiologies, a notoriously difficult area to reconstruct with local options. Fourth, all patients had critically exposed tendons and bones, requiring timely and effective coverage.

This product was chosen primarily based on its mechanism of action. Hydration is critical for exposed tendon and bone. A second consideration is a cost. The product is on the scale of hundreds of dollars at our institution. It is an easy product to apply, and it can be secured easily with local dressings or negative pressure wound therapy.

This study and the use of eHAM are not without limitations. First, eHAM is an added immediate cost for the patient. Second, this study does not directly compare eHAM to autologous grafting, human allograft, or other dermal substitutes. There is no control that shows eHAM led to more or faster healing than any of the aforementioned options. Third, this is a limited cohort of patients at a single institution. Lastly, eHAM reconstruction for this study required the patient has a donor site that can be accessed for definitive STSG coverage.

The two failures in this study reflect some of the limitations of using eHAM. Patient number 11 sustained a failure when attempting to cover a larger area of exposed bone. While no absolute sizes of exposed bone have been reported in the literature, the larger the defect the more challenging the problem. The longer bone is exposed the higher the chance of osteomyelitis and nonunion. The other patient who failed in this series, patient 14, had pitting edema in the lower extremities. The egress of fluid from the wound likely overwhelmed the eHAM and prevented the formation of a healthy bed of granulation tissue. As with other skin substitutes, eHAM is not without its complications and failures.

## CONCLUSIONS

Complex lower extremity wounds with exposed tendon or bone in a select patient population with limited reconstructive options can be successfully treated with eHAM. Thirteen of 15 patients with lower extremity wounds were salvaged in this study with the use of eHAM to treat exposed tendon and bone. This treatment option should be added to the armamentarium of the reconstructive surgeon.

## Notes

Conflicts of Interest and Sources of Funding: Medline Industries, Inc. provided financial and medical writing support for this manuscript; the choice of cases and conclusions were at the sole discretion of the authors. S.R.V. is a consultant for Medline Industries, Inc.
